# Upper Extremity Surgical Outcomes in Transgender Trauma Patients

**DOI:** 10.7759/cureus.102731

**Published:** 2026-01-31

**Authors:** Samantha Maasarani, Daniel Bahat, Christopher Jou, Kyle J Chepla

**Affiliations:** 1 Plastic Surgery, Cleveland Clinic, Cleveland, USA; 2 Plastic Surgery, MetroHealth Medical Center, Cleveland, USA

**Keywords:** outcomes, surgery, transgender, trauma, upper extremity

## Abstract

Introduction: Transgender individuals experience disproportionately high rates of mental health conditions. Previous studies have shown that psychosocial comorbidities impact recovery and outcomes after upper extremity surgery, where functional outcomes have been shown to be closely tied to pain perception and psychological well-being. Despite the increasing visibility and healthcare utilization of transgender patients, little is known about whether their postoperative outcomes differ from those of cisgender patients following upper extremity surgery. This study aimed to compare clinical and psychosocial outcomes after hand and upper extremity surgery between transgender and cisgender patients at a single tertiary-care academic institution.

Methods: A retrospective chart review of patients undergoing upper extremity surgery between 2015 and 2023 was performed. Transgender patients were identified using ICD-9/10 codes for gender dysphoria and were matched to cisgender controls.

Results: Fifty-three patients were included: 33 cisgender and 20 transgender patients. While psychiatric diagnoses were significantly more prevalent in the transgender group (71.4% vs. 35.3%, p=0.01; OR4.99, 95%CI:1.51-19.19), there were no statistically significant differences in complication rates (p=0.99), re-operation (0.19), infection (0.68), or hardware failure (0.47). Mean PHQ-9 scores were also not significantly different between groups (p=0.11).

Conclusion: Despite significantly higher psychiatric comorbidity, transgender patients undergoing upper extremity surgery demonstrated equivalent postoperative outcomes when compared to their cisgender peers. These findings suggest that transgender status alone should not be considered a risk factor for adverse outcomes in hand surgery. Ongoing work is necessary to better understand how gender identity, mental health, and access to gender-affirming care interact to shape surgical recovery.

## Introduction

Mental health disparities in the transgender population are well-documented and likely multifactorial. Transgender individuals have an elevated prevalence of associated psychiatric conditions including anxiety disorders, major depressive disorder, PTSD, and bipolar disorder, thought to result from experienced gender dysphoria, social stigma, discrimination, and limited access to gender-affirming healthcare [1-3].

Chest dysphoria in particular, a form of distress related to incongruence between one’s physical chest appearance and gender identity, is independently associated with depression and anxiety, even after adjusting for other components of gender dysphoria and social transition [2]. Conversely, gender-affirming mastectomy has been associated with significant reductions in depression (Patient Health Questionnaire (PHQ-9) scores), anxiety (General Anxiety Disorder (GAD-7) scores), and improvements in psychosocial and sexual function [4].

While the effects of gender-affirming surgeries on mental health have been studied, less is known about outcomes for transgender patients undergoing procedures unrelated to gender affirmation and the literature has only recently begun to evaluate and address this gap. A recent review of musculoskeletal care in transgender individuals emphasized the need for awareness of hormone therapy effects on healing and bone health, as well as the psychosocial and systemic barriers transgender patients face within surgical care [5]. Understanding this relationship is critically important after hand and upper extremity surgery where the relationship between psychosocial distress and postoperative outcomes has been well-established. Depression, catastrophizing, and poor pain self-efficacy have all been shown to worsen functional outcomes and prolong recovery in the early postoperative period [6-8].

We sought to evaluate whether the increased prevalence of these psychological comorbidities, which are more common in transgender patients, and known to impact outcomes after upper extremity surgery is unique in transgender patients and whether their known psychosocial vulnerabilities translate into measurable differences in clinical outcomes. We hypothesized that there would be no significant difference in complication rates or postoperative outcome measures between the two groups.

## Materials and methods

Study design and population

This IRB-approved, retrospective cohort study was conducted at a single, large, urban academic medical center. Patients who had surgery for a traumatic upper extremity injury between January 2015 and December 2023 were identified via CPT code query from the institutional electronic medical record (Appendix). Transgender patients were identified based on the presence of one or more ICD-9/10 diagnostic codes for gender dysphoria (Appendix). First, a multivariate logistic regression model analysis was performed to assess baseline patient characteristic differences between our cohorts and found only age and nicotine use status to be statistically significant. Propensity score matching was performed in a 1.5 to 1 fashion, matching cisgender patients to transgender patients by age and history of nicotine use. A nearest neighbor matching was performed where transgender patients were matched to one or more of the nearest control subjects based on their propensity score. Post-matching covariate balance was confirmed by comparing each cohort's age and nicotine use standardized differences, with values being less than 0.25. 

Data collection

Demographic variables included age, sex assigned at birth, smoking status, and medical comorbidities. For transgender patients, current hormone therapy status and prior gender-affirming surgeries (chest masculinization, chest feminization, bottom surgery, hysterectomy, orchiectomy) were recorded. Psychiatric comorbidity was defined by chart documentation of at least one mental health diagnosis, including depression, anxiety, PTSD, bipolar disorder, or personality disorders. When available, perioperative PHQ-9 scores were used as standardized measures of depressive symptoms.

Surgical data included procedure type, injury mechanism, and treatment details. Postoperative outcomes included complications including infection, hardware failure, need for hardware removal, joint stiffness, or reoperation. Complications were stratified by hormone therapy status and type of gender affirming surgery to assess potential modifying effects.

Continuous variables were compared using independent t-tests. Categorical variables were analyzed using Chi-square or Fisher’s exact tests, as appropriate. Odds ratios (OR) with 95% confidence intervals (CI) were calculated for binary outcomes. Statistical significance was defined as p < 0.05.

## Results

Demographics

A total of 53 patients met the inclusion criteria (20 transgender and 33 cisgender controls). The mean age was 46.0 years in the cisgender group and 46.6 years in the transgender group (p = 0.99), and there was no significant difference in smoking status between cohorts (p=0.43) (Table 1).

**Table 1:** Patient characteristics n: Number

	Cisgender	Transgender	p-value
	n=33	n=20	
Mean age (in years)	46	46.6	0.99
Nicotine history, n (%)			
Current	10 (30.30)	3 (15.00)	0.43
Former	4 (12.12)	1 (5.00)	
Never	11 (33.33)	8 (40.00)	
Unknown	8 (24.24)	8 (40.00)	
Psychiatric diagnosis, n (%)			
0	21 (63.64)	5 (25.00)	0.01
1	2 (6.06)	5 (25.00)	
2	5 (15.15)	6 (30.00)	
3	2 (6.06)	4 (20.00)	
4 or more	3 (9.09)	0 (0.00)	
Avg PHQ-9 score	7.091	4.091	0.11
Gender-affirming medical history, n (%)			
No hormones	–	8 (40.00)	–
Estrogen therapy	–	4 (20.00)	
Testosterone therapy	–	8 (40.00)	
Gender-affirming surgical history, n (%)			
No surgery	–	8 (40.00)	–
Chest masculinization	–	3 (15.00)	
Chest feminization	–	2 (10.00)	
Bottom surgery	–	0 (0.00)	
Hysterectomy	–	2 (10.00)	
Orchiectomy	–	1 (5.00)	

Additionally, there was no significant difference between cohorts regarding the anatomical location of injury (p=0.60) or treatment type (p=0.07) (Table 2).

**Table 2:** Traumatic hand injury characteristics n: Number

	Cisgender	Transgender	p-value
	n=33	n=20	
Anatomical location of injury, n (%)			
Distal phalanx	0 (0.00)	0 (0.00)	0.6
Middle phalanx	2 (6.06)	1 (5.00)	
Proximal phalanx	5 (15.15)	1 (5.00)	
Metacarpal	8 (24.24)	3 (15.00)	
Distal wrist	7 (21.21)	6 (30.00)	
Proximal forearm	1 (3.03)	0 (0.00)	
Proximal phalanx & metacarpal	1 (3.03)	0 (0.00)	
Proximal phalanx & distal wrist	0 (0.00)	1 (5.00)	
Distal wrist & proximal forearm	1 (3.03)	0 (0.00)	
Treatment type, n (%)			
Nonoperative	8 (24.24)	8 (40.00)	0.07
Closed reduction percutaneous pinning	6 (18.18)	2 (10.00)	
Open reduction internal fixation	16 (48.48)	10 (50.00)	
Open reduction percutaneous pinning	2 (6.06)	0 (0.00)	
External fixator	1 (3.03)	0 (0.00)	

Among the transgender cohort, eight patients (40.0%) were not on any hormone therapy, four patients (20.0%) were on estrogen therapy, and eight patients (40.0%) were on testosterone therapy (Table 1). Additionally, eight transgender patients (40.0%) did not undergo any gender-affirming surgery prior to time of injury, three patients (15.0%) had prior chest masculinization surgery, two patients (10.0%) had chest feminization surgery, zero patients had bottom-affirming surgery, two patients (10.0%) had hysterectomy, and one patient (5.0%) had an orchiectomy (Table 1).

Mental health findings

Psychiatric diagnoses were significantly more prevalent in transgender patients compared to cisgender controls (75.0% vs. 36.4%, p = 0.01), corresponding to an OR of 5.25 (95% CI: 1.53 to 18.07) (Table 1). This aligns with national data on the elevated psychiatric burden in transgender populations. PHQ-9 scores, which were available for 20 patients, showed a trend toward lower mean scores in the transgender patient group (4.091 vs. 7.091, p = 0.07), although this was not statistically significant (Table 1). 

Surgical outcomes

There was no significant difference between cisgender and transgender patients experiencing any complication postoperatively (OR 0.99, 95% CI 0.21 to 4.67, p=0.99) (Table 3). Specifically, there was also no statistically significant difference regarding infection (p=0.68), hardware failure (p=0.47), or need for re-operation (p=0.19).

**Table 3:** Outcomes by gender n: Number

	Cisgender	Transgender	p-value
	n=33	n=20	
Expected healing	28 (84.85)	17 (85.00)	0.99
Overall complication	5 (15.15)	3 (15.00)	
Reoperation	2 (6.06)	3 (15.00)	0.19
Infection	1 (3.03)	1 (5.00)	0.68
Hardware failure	2 (6.06)	2 (10.00)	0.47

Complication rates were also not affected by hormone therapy status (p=0.80), or prior gender-affirming surgery (p=0.80) (Tables 4, 5).

**Table 4:** Outcomes by gender-affirming medical treatment n: Number

	Cisgender	Transgender (n=20)		p-value
		No hormone therapy	Hormone therapy	
	n=33	n=8	n=12	
Expected healing	28 (84.85)	7 (87.50)	10 (83.33)	0.8
Complication	5 (15.15)	1 (12.50)	2 (16.67)	

**Table 5:** Outcomes by gender-affirming surgical treatment n: Number

	Cisgender	Transgender (n=20)		p-value
		No gender-affirming surgery	Gender-affirming surgery	
	n=33	n=8	n=12	
Expected healing	28 (84.85)	7 (87.50)	10 (83.33)	0.8
Complication	5 (15.15)	1 (12.50)	2 (16.67)	

## Discussion

This study demonstrates that transgender patients have similar complication rates and postoperative courses when compared to age- and comorbidity-matched cisgender controls after upper extremity surgery for traumatic injury within the limits of this small retrospective cohort, despite a higher prevalence of associated psychiatric comorbidities. This raises the possibility that psychiatric comorbidities in this patient population, which are thought to be directly related to their experienced dysphoria, may not impact surgical outcomes as seen in previous studies, and builds on prior work which found that psychological factors, such as depression and catastrophizing, negatively influence recovery and outcomes after hand surgery [6-8]. While the current study was not structured to evaluate this, it is likely a complex combination of clinical factors and social determinants, including adequate access to affirming care, appropriate pain management, and mental health resources.

One interesting finding was that, while the transgender group had a significantly higher prevalence of psychiatric conditions, their PHQ-9 scores were lower. One possible explanation is that surgical care itself, particularly in affirming or inclusive environments, may positively impact psychological well-being, even in non-gender-affirming procedures.

We hope that our findings raise awareness regarding non-gender-affirming surgical management of transgender patients. While psychosocial health is important to evaluate preoperatively for all patients, hand and upper extremity surgeons should be aware of the increased prevalence of psychiatric comorbidities in this patient population, but should not view them as a contraindication to surgery or risk for untoward outcomes. As with all patients, these issues should encourage a thoughtful, inclusive approach to care coordination and postoperative support. Finally, hand surgeons should be aware that respectful, affirming, and inclusive care environments may help support positive outcomes, regardless of psychiatric history [5]. 

While the findings for this initial study are reassuring, larger-scale studies are needed to further and better investigate nuanced outcomes using a prospective approach, patient-reported outcome measures (PROMIS scores), patient satisfaction, and more detailed measures of functional outcomes (Disability of the Arm, Shoulder and Hand (DASH)) and Michigan Hand Outcomes Questionnaire (MHQ scores). A prior scoping review on health disparities in hand surgery has highlighted the limited inclusion of gender identity in existing outcomes research, suggesting this should be a key area for future investigation [8]. Incorporation of a multicenter approach or the use of national databases to expand sample size would help to further improve power and better allow for an understanding of this unique patient experience.

The current study has several limitations. The retrospective design limited data availability for patients, and we were unable to assess injury severity or pre-injury functional status. All patients were from a single institution, which regularly provides gender affirming care and is likely associated with higher health literacy and institutional cultural competence, potentially limiting wide clinical applicability. No formal power analysis was done, and the patient groups were relatively small. Unfortunately, as a result, PHQ-9 scores were not available for all patients, and the study might have been underpowered to detect differences between the study groups or introduced a selection bias. Furthermore, no distinction was made between active or historical psychiatric diagnoses, treated versus untreated conditions, disease severity, or associated functional impairment, which could impact the interpretation and application of our findings. Finally, the use of ICD codes to identify transgender patients introduces potential for misclassification bias and may have under-identified patients and preferentially selected those with higher healthcare utilization, causing an exaggeration of mental health disparities. A prospective design using more detailed outcome measures (PROMs) would further improve generalizability and clinical applicability of our findings. Resilience is a strong predictor of recovery after upper extremity procedures, and future studies should evaluate this and pain self-efficacy alongside traditional psychological scales [8]. 

Despite these limitations, our findings challenge implicit biases that may exist in the surgical decision-making process during the care of transgender patients, with the data suggesting that transgender identity and the associated increased risk of psychiatric comorbidities alone do not portend worse surgical outcomes. The fact that transgender status did not negatively affect outcomes suggests that either these factors were effectively mitigated, experienced differently, or that these patients may have protective resilience or support mechanisms not captured in traditional metrics. 

## Conclusions

Transgender patients undergoing traumatic hand and upper extremity surgery do not experience higher rates of postoperative complications when compared to cisgender patients, even in the context of greater underlying psychiatric comorbidity. These findings emphasize the importance of equitable surgical care and counter bias that may arise from assumptions about mental health. Further prospective studies are needed to better explore functional, psychological, and patient-reported outcomes in this population.

## Appendices

**Table 6:** Billing codes used to identify traumatic upper extremity patients and gender dysphoria

Transgender	
ICD Code	Code Description
ICD-9-302.6	Gender identity disorder in children
ICD-9-302.85	Gender identity disorder in adolescents or adults
ICD-9-302.50	Trans-sexualism with unspecified sexual history
ICD-9-302.51	Trans-sexualism with asexual history
ICD_9-302.52	Trans-sexualism with homosexual history
ICD_9-302.53	Trans-sexualism with heterosexual history
ICD-10-F64.0	Transsexualism
ICD-10-F64.9	Gender identity disorder of childhood
Treatment type	
CPT Code	Code Description
CPT-26750	Closed treatment, distal phalangeal fracture, finger/thumb; w/o manipulation, each
CPT-26740	Closed treatment, articular fracture, MCP/IP joint; w/o manipulation, each
CPT-26735	Open treatment, phalangeal shaft fracture, proximal/middle phalanx, finger/thumb, w/wo fixation, each
CPT-26727	Percutaneous skeletal fixation, unstable phalangeal shaft fracture, proximal/middle phalanx, finger/thumb; w/manipulation, each
CPT-26725	Closed treatment, phalangeal shaft fracture, proximal/middle phalanx, finger/thumb; w/manipulation, each
CPT-26615	Open treatment, metacarpal fracture, single, w/wo internal/external fixation, each bone
CPT-26605	Closed treatment, metacarpal fracture, single; w/manipulation, each bone
CPT-25622	Closed treatment, carpal scaphoid (navicular) fracture; w/o manipulation
CPT-25609	Open treatment of distal radial intra-articular fracture/epiphyseal separation/inter fix of 3 or more fragments
CPT-25607	Open treatment of distal radial extra-articular fracture or epiphyseal separation, with inter fixation
CPT-25605	Closed treatment, distal radial fracture/epiphyseal separation; w/manipulation
